# Voxel-S-Value based 3D treatment planning methods for Y-90 microspheres radioembolization based on Tc-99m-macroaggregated albumin SPECT/CT

**DOI:** 10.1038/s41598-023-30824-4

**Published:** 2023-03-10

**Authors:** Gefei Chen, Zhonglin Lu, Han Jiang, Ko-Han Lin, Greta S. P. Mok

**Affiliations:** 1 Biomedical Imaging Laboratory (BIG), Department of Electrical and Computer Engineering, Faculty of Science and Technology, University of Macau, Avenida da Universidade, Taipa, Macau SAR China; 2 Ministry of Education Frontiers Science Center for Precision Oncology, University of Macau, Macau, SAR China; 3 Department of Nuclear Medicine, Taipei Veterans General Hospital, Taipei, Taiwan

**Keywords:** Radiotherapy, Targeted therapies, Cancer

## Abstract

Partition model (PM) for Y-90 microsphere radioembolization is limited in providing 3D dosimetrics. Voxel-S-Values (VSV) method has good agreement with Monte Carlo (MC) simulations for 3D absorbed dose conversion. We propose a new VSV method and compare its performance along with PM, MC and other VSV methods for Y-90 RE treatment planning based on Tc-99m MAA SPECT/CT. Twenty Tc-99m-MAA SPECT/CT patient data are retrospectively analyzed. Seven VSV methods are implemented: (1) local energy deposition; (2) liver kernel; (3) liver kernel and lung kernel; (4) liver kernel with density correction (LiKD); (5) liver kernel with center voxel scaling (LiCK); (6) liver kernel and lung kernel with density correction (LiLuKD); (7) proposed liver kernel with center voxel scaling and lung kernel with density correction (LiCKLuKD). Mean absorbed dose and maximum injected activity (MIA) obtained by PM and VSV are evaluated against MC results, and 3D dosimetrics generated by VSV are compared with MC. LiKD, LiCK, LiLuKD and LiCKLuKD have the smallest deviation in normal liver and tumors. LiLuKD and LiCKLuKD have the best performance in lungs. MIAs are similar by all methods. LiCKLuKD could provide MIA consistent with PM, and precise 3D dosimetrics for Y-90 RE treatment planning.

## Introduction

Y-90 microspheres radioembolization (RE) is an effective treatment for patients with primary and metastatic liver cancers^1^. Microspheres labeled with Y-90 are delivered to tumors through the hepatic artery and then trapped preferentially in tumoral tissue capillaries. Radiation dose delivered to tumors should be high enough to ensure therapeutic efficacy^2^, which inevitably leads to radiation to the surrounding normal tissues, possibly causing nontumoral liver (NL) complications^3^ and radiation pneumonitis^4^. Thus, to ensure both internal radiation protection and treatment efficacy, treatment planning based on pre-treatment quantitative imaging is essential to realize personalized Y-90 RE for patients^5^.

Body surface area method and partition model are recommended for injected activity (IA) calculation of resin microspheres^6,7^, while standard model and partition model are suggested for IA calculation of glass microspheres^8^. The calculation of IA by body surface area method depends on the volume of tumors and NL, and patient’s height and weight. Standard model concerns radiation dose of two compartments, i.e., liver and lungs^9^. Partition model divides the radiation-affected volume into three compartments, i.e., NL, tumors and lungs^10^. All methods require pre-treatment Tc-99m-macroaggregated albumin (MAA) planar or SPECT/CT scans to estimate the distribution of microspheres after RE. Lung shunt fraction (LSF) and tumors to normal liver ratio (TNR) should be determined from Tc-99m-MAA images and the maximum injected activity (MIA) can then be calculated based on the preset absorbed dose limits for NL and lungs for partition model. Although the three aforementioned treatment planning methods partially implement the individualized dose calculation, they only consider the mean absorbed dose and assume a uniform activity distribution throughout the compartments. On the other hand, the dose-volume histogram (DVH), calculated from voxel-level absorbed dose map, could describe the heterogeneous absorbed dose distribution within a volume-of-interest (VOI). It is frequently used in the external beam radiation therapy (EBRT) for treatment planning, and has been shown as a valuable tool for the evaluation of tumor control probability^11^ and normal tissue toxicity^12^ in Y-90 RE.

Voxel-S-Value (VSV) method is used to calculate the voxel-level absorbed dose for heterogeneous activity in a uniform medium^13^, serving as an efficient alternate to the gold standard yet time consuming Monte Carlo (MC) approach. Moreover, local energy deposition (LED), a special case of VSV, is commonly used for Y-90 absorbed dose conversion as it is a pure β^-^ emitter with shorter travelling range^14^. Several improved VSV methods for heterogeneous medium have been investigated, e.g., density correction^15^, tissue-specific VSV^16^ and center voxel scaling^17^. However, they are still compromised in absorbed dose estimation of liver-lungs interface^18^. Meanwhile, recent studies have presented 3D dosimetry treatment planning methods based on Tc-99m-MAA SPECT/CT for Y-90 RE, showing superior dosimetric results to partition model by taking inhomogeneous microspheres distributions into consideration^19–21^. These studies are based on MC, LED and standard VSV methods. Besides, the accuracy of DVH in targeted VOIs obtained by VSV methods has not been well evaluated.

In this work, we evaluated the performance of seven VSV methods, including a newly proposed tissue-specific VSV method with density correction, i.e., liver kernel with center voxel scaling and lung kernel with density correction (LiCKLuKD)^22^, on 3D dosimetrics calculations using Tc-99m-MAA SPECT/CT. Standard partition model and MC results were also compared.

## Materials and methods

### Imaging protocol and data preprocessing

Twenty sets of Tc-99m-MAA SPECT/CT patient data from University of Michigan Deep Blue Data sharing repository (n = 6)^23^ and Taipei Veterans General Hospital (n = 14) were analyzed in this study. The detailed imaging protocol for the Deep Blue Data was described in a related literature^24^. The tumor maps were available in the Deep Blue dataset, while the liver and lung maps were segmented from CT images. All images and maps were down-sampled to match with the original voxel size of SPECT images (4.8 × 4.8 × 4.8 mm^3^) for further dose calculation and analysis.

Fourteen sets of patient data from Taipei Veterans General Hospital with malignant liver tumors, who underwent both contrast enhanced CT (CECT) (MIYABI angio-CT, Siemens Healthineers, Germany) and Tc-99m-MAA SPECT/CT (Discovery NM670, GE Healthcare, USA) were retrospectively analyzed. All procedures performed in this study were approved by the Institutional Review Board of Taipei Veterans General Hospital, in accordance with the 1964 Helsinki declaration and its later amendments or comparable ethical standards. Institutional Review Board of Taipei Veterans General Hospital waived the requirement of informed consent due to the retrospective nature of this study without patient-identifiable information. All patients were administered with 111 MBq Tc-99m-MAA to simulate the Y-90 microspheres distribution for treatment planning. CECTs were collected for segmentation of tumors with a voxel size of 0.68 × 0.68 × 5.00 mm^3^ and a matrix size of 512 × 512 × 44, covering the whole liver and partial bottom lungs. Sixty SPECT projections covering the whole liver and whole lungs were acquired with a low-energy high-resolution collimator over 360°, with a primary window of 126–154 keV and a scatter window of 114–126 keV. These projections were then reconstructed with ordered subset expectation maximization (2 iterations, 10 subsets), incorporating CT-based attenuation correction and dual-energy window-based scatter correction. The SPECT reconstruction voxel size is 4.42 × 4.42 × 4.42 mm^3^ and the matrix size is 128 × 128 × 128. Corresponding low-dose CT (LDCT) data covering whole liver and whole lungs were acquired (120 kV, 57 mA) during shallow free breathing, with a reconstructed voxel size of 0.98 × 0.98 × 3.75 mm^3^ and a matrix size of 512 × 512 × 108.

The tumor map was segmented in CECT by an experienced radiologist due to its distinct tumor contours. CECT and tumor map were aligned to LDCT by rigid registration using Elastix^25^. Then, VOIs of whole liver and whole lungs were manually delineated in LDCT. The NL contours were obtained by excluding the tumors from the whole liver. Finally, LDCT and VOI maps, i.e., liver, lungs and tumors, were downsampled to match with the voxel size of SPECT images for further dose calculation and analysis.

**Figure 1:**
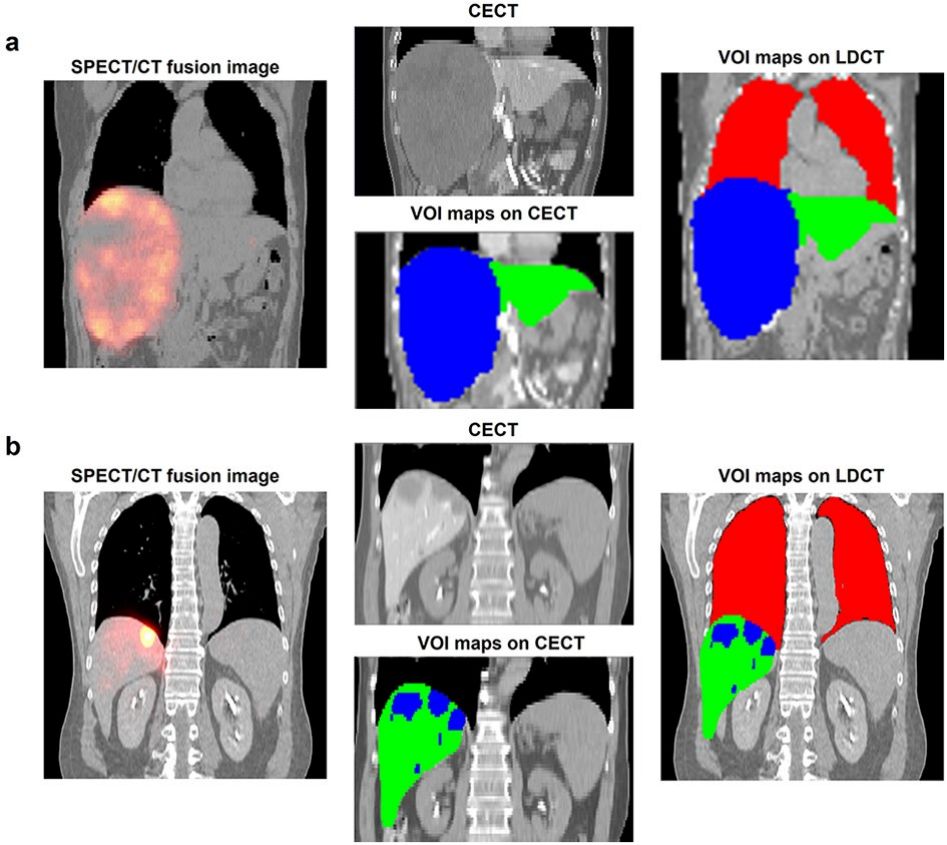
Two sample patients with (**a**) a single tumor and (**b**) multiple tumors. Red: lungs, blue: tumors, green: NL.

SPECT/CT fusion images, CECT and VOI binary maps of two sample patients were shown in Fig. 1. SPECT and corresponding CT images were all well aligned in this study. LDCT images were converted to density maps and tissue maps for MC- and VSV-based absorbed dose conversion^26^. Activity in each voxel (*A*) was obtained through a self-calibration factor defined as the (total injected activity)/(counts in the SPECT images covering whole liver and lungs)^27^. The time-integrated activity (TIA) images $\widetilde{A}$ were generated assuming the microspheres were all trapped in tumors with only physical decay afterwards:

1 $$\tilde{A}_{{Voxel}} = \frac{{A_{{Voxel}} }}{\lambda }$$

where $${{\uplambda }} = \text{ln}2/\text{T}_{1/2}$$, $${\text{T}}_{1/2} = 64.04\,{\text{h}}$$ for Y-90.

### MC simulation and VSV generation

MC simulation and VSV generation were performed using GATE v.9.0, which has been validated in Y-90 dosimetry previously^28^. Y-90 emitter was simulated by defining an ion source. The physics list emstandard_opt4 was used in the simulation, including photoelectric effect, Compton scattering, Rayleigh scattering, multiple scattering and pair production modeled for photons, while positron annihilation, ionization and bremsstrahlung effect were modeled for β particles. Cutting energy of 10 keV was set for all particles to reduce the simulation time. Density map and tissue map were input for creating the voxelized phantom, and TIA images were read in as the voxelized source*.* A total number of 10^10^ primary decays were simulated. The statistical uncertainty of mean absorbed dose by MC simulation is 0.02% for liver and 0.78% for lungs. DoseActor was attached to the voxelized phantom to collect the 3D absorbed dose distribution. VSVs for lungs (*ρ*_*Lung*_ = 0.26 g/cm^3^) and liver (*ρ*_*Liver*_ = 1.06 g/cm^3^) were also generated using GATE, with the same voxel size as the two sets of SPECT images used in this study and a matrix size of 21 × 21 × 21 and 5 × 5 × 5 respectively, to include > 99% released energy. A total of 10^9^ primaries were set for all VSV simulations to ensure uncertainties < 0.01%. To speed up the simulation, all simulations were split into 64 subsets with different random seeds for parallel running in the cluster with four Xeon 6248 CPUs and 128 GB RAM.

### VSV-based absorbed dose conversion

Local energy deposition (LED) method requires the mean energy of β particles and the mass of each voxel for voxel-level dose calculations, serving as a special case of VSV in this study:

2 $${D_{{Voxel}} = \tilde{A}_{{Voxel}} \times \frac{E}{M}}$$

where *D*_*Voxel*_ is the voxel dose of a 3D absorbed dose map, is the multiplication operator, *E* is the mean energy of Y-90, i.e., 49.67 (J/GBq), and *M* is the mass of a voxel.

Other six VSV methods are evaluated in this study:

1. Liver kernel (LiK)^13^:
  3 $${D = \tilde{A} \otimes K_{{Liver}} }$$
  where is the convolution operator, $\widetilde{A}$ is the whole body TIA map and ${K}_{Liver}$ is the liver VSV.
2. Liver kernel for liver TIA and lung kernel for lung TIA (LiLuK)^16^
  4 $${D = \tilde{A}_{{Liver}} \otimes K_{{Liver}} + \tilde{A}_{{Lungs}} \otimes K_{{Lung}} }$$
  where ${\widetilde{A}}_{Liver}$ is the liver TIA map, ${\widetilde{A}}_{Lung}$ is the lung TIA map and ${K}_{Lung}$ is the lung VSV.
3. Liver kernel with density correction (LiKD)^15^
  5 $$D = \tilde{A} \otimes K_{{Liver}}^{D}$$
  where ${{K}_{ }}_{Liver}^{D}\left(i,j,k\right)={K}_{Liver}\left(i,j,k\right)\frac{{\rho }_{Liver}}{\rho \left(i,j,k\right)}$, $\rho \left(i,j,k\right)$ indicates the density of voxel (i, j, k).
4. Liver kernel with center voxel scaling (LiCK)^17^
  6 $$D = \tilde{A} \otimes K_{{Liver}}^{C}$$
  where ${K}_{Liver}^{C}={K}_{Liver}\frac{{\rho }_{Liver}}{{\rho }_{center voxel}}$ and ${\rho }_{center voxel}$ means the voxel density corresponding to the voxel where the VSV center locates during the convolution.
5. Liver kernel and lung kernel with density correction (LiLuKD)^29^
  7 $$D = \tilde{A}_{{Liver}} \otimes K_{{Liver}}^{D} + \tilde{A}_{{Lungs}} \otimes K_{{Lung}}^{D} K$$
  where $${{K}_{ }}_{Lung}^{D}\left(i,j,k\right)={K}_{Lung}\left(i,j,k\right)\frac{{\rho }_{Lung}}{\rho \left(i,j,k\right)}$$.
6. Newly proposed LiCKLuKD
  8 $$D = \tilde{A}_{{Liver}} \otimes K_{{Liver}}^{C} + \tilde{A}_{{Lungs}} \otimes K_{{Lung}}^{D}$$

### Dosimetry assessment

The accuracy of ${D}_{NL}^{mean}$, ${D}_{Tumors}^{mean}$ and ${D}_{Lungs}^{mean}$ obtained by partition model and VSV methods were evaluated against MC results. The DVH dosimetrics of NL, tumors, and lungs generated by VSV methods were also evaluated with MC results. All dosimetric calculations were based on an IA of 3 GBq, which is the MIA suggested by the empirical method^30^ and semi-empirical body surface area method^31^. Other dosimetrics commonly used in EBRT^32^ were also evaluated for VSV methods and compared with MC results: percentage of the volumes receiving at least 70 Gy for NL (V_NL, 70 Gy_), 200 Gy for tumors (V_Tumors, 200 Gy_), 5 Gy (V_Lungs, 5 Gy_) and 13 Gy (V_Lungs, 13 Gy_) for lungs.

To evaluate the DVH accuracy, the mean absolute error (MAE) of the differential DVH in each VOI was calculated for VSV methods as compared to MC according to Eq. 9^33^, based on absorbed dose bins of 1 Gy:

9 $$MAE = \frac{1}{{{\text{maximum absorbed dose}}}}\sum\limits_{{k = 1}}^{{{\text{maximum absorbed dose}}}} {|V_{{VOI,{{}}k}}^{{VSV}} - V_{{VOI,{{}}k}}^{{MC}} )|}$$

where *k* is the absorbed dose bin index, and VOI includes tumors, NL and lungs. Cumulative DVH is also generated for the sample patients.

### Injected activity assessment

Assuming that Y-90 microspheres and Tc-99m-MAA have the same distribution, MIA is determined by considering the upper absorbed dose limit of lung and NL absorbed dose. For partition model, the LSF and TNR values need to be measured from Tc-99m-MAA planar or SPECT images.

10 $$LSF\left( \% \right) = 100\% \times \frac{{A_{{Lungs}} }}{{A_{{Lung}} + A_{{Liver}} }}$$

where *A*_*Lungs*_ is the activities of the lungs and *A*_*Liver*_ is the activities of the whole liver.

11 $${\text{TNR}} = \frac{{A_{{Tumors}} /M_{{Tumors}} }}{{A_{{NL}} /M_{{NL}} }}$$

where ${A}_{Tumors}$*,* ${A}_{NL}$ and ${M}_{Tumors}$*,* ${M}_{NL}$ are the activities and mass of tumors and NL, respectively. Mass for partition model is calculated based on the volumes measured from CT and assuming a uniform density of liver (1.06 g/cm^3^) and lungs (0.26 g/cm^3^).

MIA can be calculated from the following equation:

12 $$MIA_{{PM}} = \min \left[ {\frac{{(M_{{Tumors}} \times TNR + M_{{NL}} ) \times D_{{NL}}^{{max}} }}{{49.67 \times \left( {1 - LSF/100} \right)}},\frac{{M_{{Lungs}} \times D_{{Lungs}}^{{max}} }}{{49.67 \times LSF/100}}} \right]$$

where ${D}_{NL}^{max}$ and ${D}_{Lungs}^{max}$ is the upper limit of NL and lungs absorbed dose respectively, and usually set to 70 Gy and 30 Gy in partition model, while 49.67 (J/GBq) is the constant converting TIA to absorbed dose.

The MIA for the 3D dosimetric models is obtained as follows:

13 $$MIA_{{3D}} = {\text{min}}\left[ {\frac{{D_{{NL}}^{{max}} }}{{D_{{NL}}^{{mean}} /IA_{{MAA}} }},\frac{{D_{{Lungs}}^{{max}} }}{{D_{{Lungs}}^{{mean}} /IA_{{MAA}} }}} \right]$$

${D}_{NL}^{max}$ is 70 Gy and ${D}_{Lungs}^{max}$ is 30 Gy. ${D}_{VOI}^{mean}$ is the mean absorbed dose calculated by MC and VSV. and IA_MAA_ is 111 MBq for data from Taiwan and 222 MBq for Deepblue data. MIA calculated by partition model and VSV methods were compared with MC results.

The two-tailed paired samples Wilcoxon test with Bonferroni correction was performed between results of MC and other methods for mean dose and DVH dosimetrics, and a p value of ≤ 0.05 indicates statistical significance. Methods with superior performance in all evaluated indices, particularly with no significant difference in ${D}_{NL}^{mean},$ ${D}_{Tumors}^{mean}$, and ${D}_{Lungs}^{mean}$ as compared to MC were selected as the best approaches.

## Results

### Patient and tumor characteristics

The patient characteristics are listed in Table 1. In total, 77 tumors were segmented and analyzed with volume ranging from 12.4 to 1948.2 mL. The NL and lungs volumes ranged from 484.7 to 1900.6 mL and 1958.4 to 3553.9 mL, respectively. The mean density of liver and lung ranges from 1.05 to 1.09 and 0.20 to 0.35 g/cm^3^, respectively. The TNR and LSF were calculated from Tc-99m-MAA SPECT/CT images for partition model, ranging from 1.3 to 38.9 and 1.5% to 21.2%, respectively.

**Table 1:** Patient characteristics.

Patient no.	Microsphere type	LSF (%)	TNR	Tumors vol. (mL)	NL vol (mL)	Liver density (g/cm^3^)	Lungs vol (mL)	Lung density (g/cm^3^)
1	Glass	1.5	1.3	20.6	1372.8	1.07 ± 0.01	3591.2	0.27 ± 0.19
2	Glass	8.0	38.9	58.3	1311.1	1.08 ± 0.01	3168.5	0.31 ± 0.21
3	Glass	2.5	15.0	19.26	1787.8	1.07 ± 0.02	1958.4	0.32 ± 0.21
4	Glass	2.1	2.8	71.58	1589.3	1.07 ± 0.01	2701.3	0.29 ± 0.20
5	Glass	2.9	4.2	24.70	1628.6	1.05 ± 0.02	2666.2	0.32 ± 0.21
6	Glass	6.6	3.1	95.94	1276.7	1.07 ± 0.02	3553.9	0.25 ± 0.23
7	Resin	13.7	3.6	1930.0	1033.5	1.07 ± 0.05	3297.3	0.25 ± 0.20
8	Resin	5.8	8.1	183.6	1359.5	1.07 ± 0.06	3144.4	0.31 ± 0.23
9	Resin	2.7	1.7	318.3	999.6	1.07 ± 0.09	2631.9	0.29 ± 0.23
10	Resin	3.2	4.7	1948.2	1483.5	1.08 ± 0.05	2258.3	0.27 ± 0.20
11	Resin	7.3	2.2	857.3	1086.2	1.07 ± 0.07	3022.3	0.23 ± 0.17
12	Resin	2.2	4.2	12.4	1019.4	1.08 ± 0.08	2331.7	0.26 ± 0.18
13	Resin	1.7	2.3	129.5	1187.9	1.08 ± 0.06	2379.9	0.25 ± 0.18
14	Resin	1.6	2.4	285.6	1900.6	1.08 ± 0.07	3791.4	0.22 ± 0.15
15	Resin	17.8	17.3	319.4	700.9	1.10 ± 0.02	3754.4	0.22 ± 0.17
16	Resin	4.8	1.6	228.3	1164.1	1.08 ± 0.02	3774.8	0.20 ± 0.14
17	Glass	4.6	6.3	152.1	1671.7	1.09 ± 0.03	2682.7	0.35 ± 0.17
18	Resin	8.2	3.9	685.0	1040.2	1.09 ± 0.03	2718.6	0.31 ± 0.20
19	Resin	21.2	2.5	899.7	484.7	1.09 ± 0.02	2851.8	0.24 ± 0.17
20	Resin	6.7	1.2	1329.1	1280.3	1.08 ± 0.02	2090.3	0.34 ± 0.14

*LSF* lung shunt fraction, *TNR* tumor to normal liver ratio, *NL* non-tumoral liver.

### Mean absorbed dose analysis

The mean absorbed dose difference of partition model and VSV methods compared with MC are reported in Table 2, and the separate results for two datasets are listed in Tables S1 and S2. For ${D}_{NL}^{mean}$, LiLuK has the largest deviation of 3.03%, while LiCKLuKD (0.18%) and LiKD (0.23%) have the smallest deviation followed by LiLuKD (0.27%). For ${D}_{Tumors}^{mean}$, partition model has the largest deviation (6.36%) among all patients followed by LED and LiLuK. LiLuKD has the smallest deviation followed by LiKD and LiCKLuKD. For ${D}_{Lungs}^{mean}$, LiCKLuKD has the best agreement with MC result followed by LiLuKD and LiCK. Partition model performs better than the remaining VSV methods and LiK has the poorest performance. Methods with lung kernel or density correction, e.g., LiCKLuKD, LiKD and LiLuK, have improved performance in lungs. Table 3 shows statistical analysis results of different dose conversion methods as compared to MC. The statistical analysis shows that LiK, LiLuK and LiCK have significant differences with MC results in ${D}_{NL}^{mean}$. LiKD, LiLuCK and LiCKLuKD have no significant difference with MC results in ${D}_{Tumors}^{mean}$. LED, LiK, LiKD and LiCK have significant differences with MC results in ${D}_{Lungs}^{mean}$.

**Table 2:** Absolute percent differences of ${D}^{mean}$ in VOIs using partition model and VSV methods compared with MC.

Methods	Partition model	LED	LiK	LiLuK	LiKD	LiCK	LiLuKD	LiCKLuKD
${D}_{NL}^{mean}$	1.13 ± 1.42 [0.14, 4.20]	0.97 ± 1.24 [0.01, 3.92]	1.65 ± 0.92 [0.54, 3.90]	3.03 ± 1.40 [0.36, 6.55]	0.23 ± 0.16 [0.01, 0.59]	0.76 ± 0.57 [0.40, 2.74]	0.27 ± 0.16 [0.03, 0.66]	0.18 ± 0.14 [0.02, 0.54]
${D}_{tumors}^{mean}$	2.63 ± 1.65 [0.43, 6.36]	2.59 ± 1.62 [0.40, 6.16]	1.39 ± 0.85 [0.00, 3.17]	2.00 ± 0.92 [0.57, 3.25]	0.58 ± 1.22 [0.02, 5.45]	1.06 ± 1.16 [0.43, 5.52]	0.55 ± 1.21 [0.02, 5.45]	0.64 ± 1.13 [0.02, 4.98]
${D}_{lungs}^{mean}$	8.53 ± 6.52 [0.17, 19.54]	18.48 ± 7.75 [2.70, 34.57]	72.20 ± 4.76 [64.31, 79.05]	18.07 ± 11.22 [0.18, 34.98]	25.02 ± 7.94 [11.70, 42.00]	17.94 ± 6.60 [4.31, 30.62]	9.65 ± 5.59 [0.62, 18.40]	4.86 ± 3.21 [0.79, 15.64]

Mean ± std, [min, max].

**Table 3:** Statistical analysis results of different VSV and LED dosimetric results as compared to MC.

Methods	Partition model	LED	LiK	LiLuK	LiKD	LiCK	LiLuKD	LiCKLuKD
${D}_{NL}^{mean}$	0.4536	0.2616	**0.0056**	** < 0.0001**	> 0.9999	**0.0040**	0.4536	0.6664
${D}_{tumors}^{mean}$	** < 0.0001**	** < 0.0001**	** < 0.0001**	** < 0.0001**	0.8480	** < 0.0001**	> 0.9999	0.0752
${D}_{lungs}^{mean}$	> 0.9999	** < 0.0001**	**0.008**	> 0.9999	** < 0.0001**	** < 0.0001**	0.3968	> 0.9999
${V}_{NL, 70 Gy}$	N/A	** < 0.0001**	0.2324	** < 0.0001**	0.4284	> 0.9999	**0.0287**	> 0.9999
${V}_{\mathrm{tumors}, 200 Gy}$	N/A	**0.0427**	**0.0077**	** < 0.0001**	> 0.9999	> 0.9999	> 0.9999	> 0.9999
${V}_{\mathrm{lungs}, 13 Gy}$	N/A	> 0.9999	** < 0.0001**	0.1407	> 0.9999	> 0.9999	> 0.9999	> 0.9999
${V}_{\mathrm{lungs}, 5 Gy}$	N/A	**0.0014**	** < 0.0001**	**0.0210**	> 0.9999	> 0.9999	> 0.9999	> 0.9999

Significant values are in bold.

### 3D dosimetrics comparison

**Figure 2:**
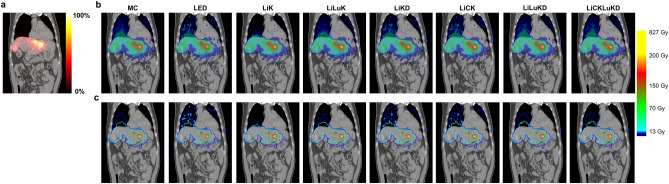
Coronal view of (**a**) the SPECT/CT fusion image, (**b**) the absorbed dose map and (**c**) isodose contour of a sample patient for MC and different VSV methods.

Isodose curves and absorbed dose map of a sample patient by MC and VSV methods are shown in Fig. 2. The absorbed dose distributions in liver and tumors are similar for all methods while more a pronounced difference is observed in lungs. Stomach, right kidney and gallbladder also receives radiation from the Y-90 microspheres in liver potentially. The DVH dosimetrics calculated by MC and deviations of VSV generated DVH dosimetrics with MC are reported in Table 4, and the separate results for two datasets are listed in Tables S3 and S4. For ${V}_{\mathrm{NL}, 70 Gy}$, LiLuK has the largest deviation of 3.03%, while LiKD, LiCK, LiLuKD and LiCKLuKD have better agreement with MC. LED has the largest deviation of 29.29% in ${V}_{\mathrm{Tumors}, 200 Gy}$, and LiKD, LiCK, LiLuKD and LiCKLuKD have mean deviation < 1.1%. The ${V}_{\mathrm{Lungs}, 13\mathrm{ Gy}}$ and ${V}_{\mathrm{Lungs}, 5\mathrm{ Gy}}$ results are similar. LiK has the largest deviation, while LiLuKD and LiCKLuKD have the smallest deviation. Except for LED, LiLuK and LiCK, other VSV methods have no significant difference with MC in ${V}_{NL, 70 Gy}$. LED, LiK, and LiLuK have significant differences with MC in ${V}_{Tumors, 200 Gy}$. For ${V}_{Lungs, 13 Gy}$, only LiK has significant differences with MC, while for ${V}_{Lungs, 5 Gy}$, LED, LiK and LiLuK have significant differences with MC.

**Table 4:** Absolute percent differences of DVH dosimetrics using VSV methods compared with MC and the dosimetrics calculated by MC.

Methods	MC	LED	LiK	LiLuK	LiKD	LiCK	LiLuKD	LiCKLuKD
${V}_{NL, 70 Gy}$	30.09 ± 17.57 [3.43, 58.36]	2.08 ± 1.74 [0.02, 6.01]	1.62 ± 1.32 [0.17, 4.94]	3.03 ± 2.02 [0.27, 8.21]	0.24 ± 0.22 [0.03, 0.99]	0.18 ± 0.22 [0.00, 0.86]	0.30 ± 0.2 [0.00, 0.99]	0.19 ± 0.15 [0.00, 0.51]
${V}_{\mathrm{tumors}, 200 Gy}$	43.86 ± 33.89 [0.00, 92.85]	1.60 ± 6.86 [0.00, 29.29]	3.74 ± 5.52 [0.00, 23.91]	4.10 ± 5.48 [0.00, 23.91]	0.83 ± 1.36 [0.00, 5.88]	0.51 ± 0.62 [0.00, 2.02]	0.79 ± 1.36 [0.00, 5.88]	0.42 ± 0.61 [0.00, 2.26]
${V}_{\mathrm{lungs}, 13 Gy}$	8.90 ± 8.08 [0.55, 39.61]	13.31 ± 32.24 [2.30, 111.63]	75.25 ± 8.46 [61.22, 96.19]	21.10 ± 19.08 [0.45, 70.21]	24.88 ± 29.45 [0.27, 111.61]	24.24 ± 27.39 [1.34, 106.76]	13.74 ± 19.59 [0.08, 89.19]	12.82 ± 18.88 [0.73, 86.61]
${V}_{\mathrm{lungs}, 5 Gy}$	23.09 ± 20.41 [5.86, 81.44]	17.30 ± 9.66 [0.45, 35.99]	67.93 ± 11.11 [55.52, 95.78]	14.30 ± 10.27 [1.95, 35.99]	11.95 ± 8.33 [0.53, 28.15]	11.07 ± 7.80 [0.11, 27.81]	4.76 ± 4.90 [0.38, 16.15]	5.88 ± 4.78 [0.09, 17.10]

Mean ± std, [min, max].

**Figure 3:**
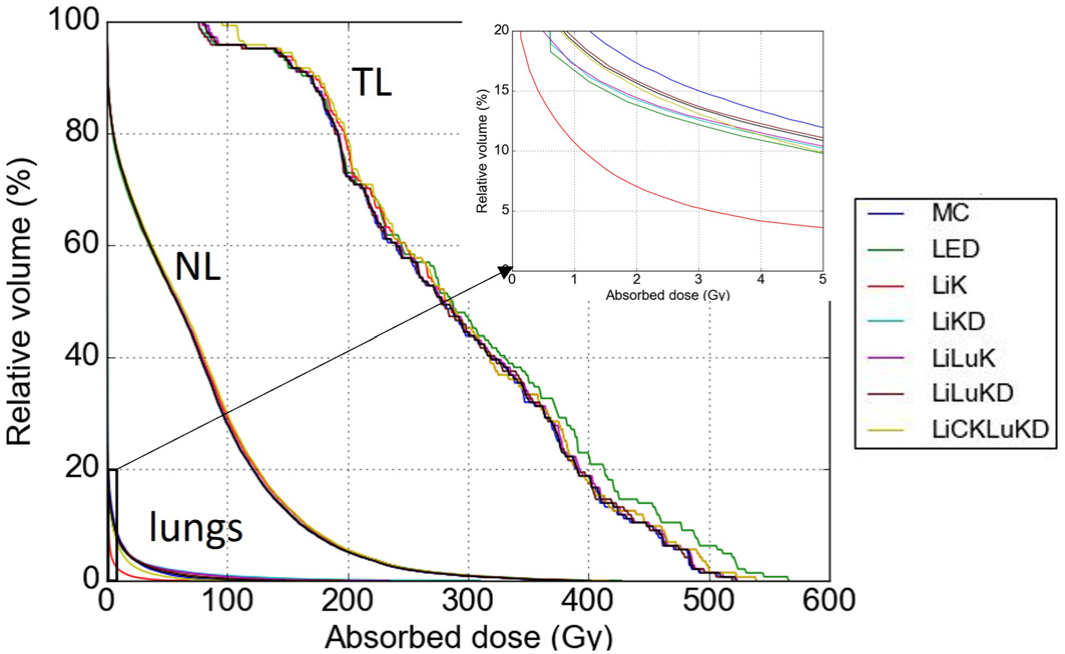
Cumulative DVH for NL, tumors, lungs and magnified image of lungs by MC and VSV methods for another sample patient.

The cumulative DVH curves of another sample patient for NL, tumors and lungs are shown in Fig. 3. The volume fraction for absorbed dose > 70 Gy in NL takes up ~ 50%. All VSV methods are close to MC in NL. For tumors, LED overestimates the volume fraction in the high absorbed dose region, i.e., ~ 230 to 520 Gy. For lungs, the volume fraction > 5 Gy is about 13% for this patient. LiK has substantially underestimated the volume fraction. As expected, tumors have higher absorbed dose as compared to NL, while lungs have the lowest absorbed dose.

The MAE of differential DVH is reported in Table 5. LiLuK, LED and LiK have the maximum MAE in NL, tumors and lungs, respectively. LiKD, LiCK and LiCKLuKD have lower MAE in NL and tumors. The MAE of LiLuKD is the lowest in lungs followed by LiCKLuKD. The execution time of all VSV methods takes up within 10 s using fast Fourier transform.

**Table 5:** MAE of differential DVH by VSV methods in different VOIs compared with MC.

VSV methods	LED	LiK	LiLuK	LiKD	LiCK	LiLuKD	LiCKLuKD
NL	28.92	20.05	35.74	4.10	4.59	8.37	2.98
Tumors	202.16	98.53	133.07	31.35	30.79	47.25	27.22
Lungs	36.51	77.14	22.12	38.02	38.02	8.37	11.12

### MIA comparison

**Figure 4:**
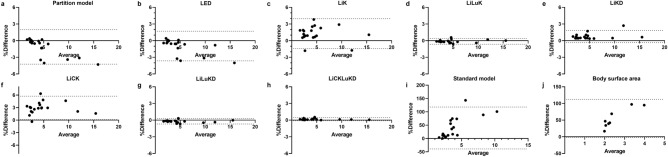
Bland–Altman plots with 95% confidence interval (CI) of MIA differences (%) between MC and other methods. The dashed lines represent 95% CI of mean differences.

Figure 4 shows the Bland–Altman plots of MIA results between MC and other methods. The restriction of lung absorbed dose was not applied for MIA calculation as ${D}_{Lungs}^{mean}$ of all patients is well < 30 Gy. The MIA calculated by MC are regarded as the baseline, ranging from 1.67 to 15.51 GBq. Partition model and VSV methods except for LiCK have a deviation of < 5% in MIA calculation compared with MC.

## Discussion

The MIA of Y-90 RE is usually based on conventional 2D models such as partition model and body surface area method currently in the clinic. However, these methods do not consider the heterogeneity of microspheres distribution. Some groups also proposed 3D treatment planning method for Y-90 RE. Morán et al.^21^ compared different dose conversion methods, standard partition model, partition model considering multiple tumors, LED and LiK. Dieudonné et al.^19^ proposed a 3D dose conversion method for treatment planning by LiK. However, both of them did not consider potential variations in lung absorbed dose estimation and did not compare their results with MC simulations. Petitguillaume et al.^20^ used the DVH dosimetrics by MC simulations for Y-90 RE treatment planning but not for VSV. In this study we show that VSV is an alternative to partition model and MC in MIA calculation, and is feasible to provide accurate and complete DVH dosimetrics for Y-90 RE treatment planning in real time.

VSV methods could provide slightly better estimation on ${D}_{Tumors}^{mean}$ than partition model and the performance of VSV methods are improved by applying density corrections. LiK has similar results with partition model on ${D}_{NL}^{mean}$ and ${D}_{Tumors}^{mean}$ consistent with Dieudonné et al.^19^. LED does not consider the cross-voxel radiation, with a deviation of 6.16% for tumors. Partition model, LED and LiK have larger difference of ${D}_{Tumors}^{mean}$ than ${D}_{NL}^{mean}$, consistent with the results of Morán et al.^21^. Hashikin et al.^34^ shows more pronounced absorbed dose difference of tumors and NL in partition model as compared to MC in phantom simulations. This difference in real patients may be less obvious due to the tumor VOIs may be generally larger on SPECT to compensate for the partial volume effect, thus alleviating the errors of partition model in not modeling the cross-radiation effect. Tumors and NL possess similar density thus their dosimetric accuracy is expected to be similar. Our results show that the error is slightly higher for tumors, which could be attributed to the more pronounced partial volume effect for tumors. The limit of ${D}_{Tumors}$ are not considered in IA calculation currently, as functions of the critical organs are of primarily concern to minimize toxicity. However, tumor dosimetry would be of interest as an indicator for tumor response^35–37^.

The lung absorbed dose errors are much larger than tumors and NL in general, as activity from liver has potential contributions and cannot be adequately modeled from some evaluated methods. The lung tissue is also quite heterogeneous, with densities range from 0.00126 g/cm^3^ (air) to 1.04 g/cm^3^ (soft tissue), posing significant problems for methods without density corrections. LiK has the largest deviation in ${D}_{Lungs}^{mean}$ due to the large difference of density between liver and lung medium. The assumed densities of liver and lungs are close to the mean liver and lungs density of different patients in this study, verifying the accuracies of VSV kernels generated in the assumed liver and lung media. The usage of lung kernel, e.g., LiLuK, could improve ${D}_{Lungs}^{mean}$ estimation, consistent with results of Mikell et al.^9^. LiCK has higher errors in ${D}_{NL}^{mean}$ and ${D}_{Tumors}^{mean}$ than LiKD, and less errors in ${D}_{Lungs}^{mean}$, consistent with results from Götz et al.^17^. Thus, a combination of center voxel scaling and density correction could further improve the performance of LiLuK and LiLuKD, i.e., LiCKLuKD as proposed in this study, which has better performance in lungs.

The DVH dosimetrics results are similar to mean absorbed dose. The VSV methods with corrections, i.e., LiKD, LiLuKD and LiCKLuKD, could achieve < 5.88% deviation of V_NL, 70 Gy_ and V_Tumors, 200 Gy_. In addition, both LiLuKD and LiCKLuKD have < 14% mean deviation for V_Lungs, 13 Gy_ and < 5% mean deviation for V_Lungs, 5 Gy_, respectively, outperforming other VSV methods. However, ${D}_{Lungs}^{mean}$ was more relevant than DVH of lungs in predicting clinically significant radiation pneumonitis in external beam radiotherapy^38^. LSF is also considered to be correlated with radiation pneumonitis in Y-90 RE^39^. Thus, the DVH of the lungs may be not a critical factor for determining MIA in Y-90 RE. The performances of VSV methods on MAE are consistent with results of the DVH dosimetrics. LiKD, LiCK, LiLuKD and LiCKLuKD have smaller deviation on NL and tumors. LiCKLuKD has the smallest deviation of DVH of lungs.

The calculation of MIA depends on ${D}_{NL}^{mean}$ and ${D}_{Lungs}^{mean}$, though the prescription is usually bounded by ${D}_{NL}^{mean}$ instead of ${D}_{Lungs}^{mean}$ as the later dose limit is usually not reached. However, patients are commonly excluded for treatment if their LSF > 20%. This criteria may not be translated to ${D}_{Lungs}^{mean}$ >30 Gy as shown in this study (Patient 19, LSF = 21.2%, ${D}_{Lungs}^{mean}=16.07$ Gy by MC), while ${D}_{Lungs}^{mean}$ of Patient 2 is > 20 Gy with LSF = 8.0%. Thus, LSF > 20% may not be a good criteria for excluding patients from Y-90 RE, as indicated by the latest user manual^6^. The MIA difference between VSV and partition model is negligible, consistent with Dieudonné et al.^19^, while Petitguillaume et al.^20^ observed a bigger difference. The discrepancy could be attributed to the difference in activity calibration method. They also showed that the MIA could be further increased based on DVH dosimetrics instead of mean absorbed dose, yet the clinical application of the DVH dosimetrics still needs to be further studied. Notably, the partition model has comparable performance with MC in MIA and other VSV methods in MIA. Thus, its clinical effectiveness is justified especially considering its relatively simple implementation. However, VSV methods can be used to replace partition model for MIA calculation in the clinics, with the advantage of providing further 3D dosimetric information. Considering the real-time execution, relatively small dosimetrics errors, and with no significant difference as compared with MC in mean dose of NL, tumors and lungs, LiLuKD and LiCKLuKD are recommended for absorbed dose conversion.

We implemented the density correction (Eq. 5) differently as compared to ref 14, to be consistent with the center voxel scaling (Eq. 6) implementation. Both should yield the same results as the convolution is a linear operator. The accuracy of VSV methods using tissue specific kernels depends on the segmentations of liver and lungs, which are expected to be less subjected to errors based on CT data. However, mismatches between emission imaging data and the corresponding CT also impact VSV-based dosimetry, and are evaluated in our previous studies^40–42^. Registrations between CT and emission imaging data will ease this concern^43^. To the best of our knowledge, this is the first study to systematically analyze different VSV methods for Y-90 RE based on Tc-99m SPECT/CT. One limitation of our study is that the distributions of pre-treatment Tc-99m SPECT images and post-treatment Y-90 PET or SPECT images may not be exactly the same and could be affected by many factors, e.g., catheter positioning, particle size, liver and lungs volume^44^. The performance of VSV methods on post-therapy voxel level dosimetry, i.e., Y-90 microshere SPECT and Y-90 microsphere PET, is investigated in another study from our group, where LiLuKD and LiCKLuKD still demonstrate superior performance as compared to other methods^22^.

## Conclusion

Seven VSV methods were evaluated for the 3D absorbed dose conversion in 20 patients with pre-treatment Tc-99m-MAA SPECT/CT images for Y-90 RE, along with partition model and MC. VSV methods with effective correction and tissue-specific kernel, i.e., LiLuKD and our proposed LiCKLuKD, could provide MIA consistent with partition model, and additional precise DVH dosimetrics for potential improved treatment planning considering radiobiological effects.

## Supplementary Information

Supplementary Information.

## Data Availability

The first datasets analyzed during the current study are available in the University of Michigan—Deep Blue Data repository, [https://doi.org/10.7302/pf4m-vn04]. The second data analyzed during the current study are not publicly available due to ethical requirements for hospitals but are available from the corresponding author on reasonable request.

## Abbreviations

CECT: Contrast enhanced CT
CI: Confidence interval
DVH: Dose-volume histogram
EBRT: External beam radiation therapy
IA: Injected activity
LED: Local energy deposition
LDCT: Low-dose CT
LiCK: Liver kernel with center voxel scaling
LiCKLuKD: Liver kernel with center voxel scaling and lung kernel with density correction
LiK: Liver kernel
LiKD: Liver kernel with density correction
LiLuK: Liver kernel and lung kernel
LiLuKD: Liver kernel and lung kernel with density correction
LSF: Lung shunt fraction
MAA: Macroaggregated albumin
MC: Monte Carlo
MIA: Maximum injected activity
NL: Nontumoral liver
RE: Radioembolization
TNR: Tumor to normal liver ratio
VOI: Volume-of-interest
VSV: Voxel-S-Value
