# Supplementary material for: Voxel-S-Value based 3D treatment planning methods for Y-90 microspheres radioembolization based on Tc-99m-macroaggregated albumin SPECT/CT
Source: Sci Rep. 2023 Mar 10;13:4020. doi: 10.1038/s41598-023-30824-4 (PMC10006243; doi:10.1038/s41598-023-30824-4)
Supplement: Supplementary file 1 — Supplementary Information. [file 41598_2023_30824_MOESM1_ESM.docx]

Table S1 Absolute percent differences of $D^{mean}$ in VOIs using partition model and VSV methods compared with MC in patient data of University of Michigan Deep Blue Data sharing repository (n=6).

| Methods | Partition model | LED | LiK | LiLuK | LiKD | LiCK | LiLuKD | LiCKLuKD |
| --- | --- | --- | --- | --- | --- | --- | --- | --- |
| $D_{NL}^{mean}$ | 0.76  [0.27, 2.41] | 0.69  [0.10, 2.90] | 0.96  [0.60, 1.76] | 2.07  [0.34, 5.09] | 0.13  [0.08, 0.47] | 0.50  [0.49, 0.56] | 0.16  [0.08, 0.56] | 0.14  [0.07, 0.26] |
| $D_{TL}^{mean}$ | 3.73  [0.64, 4.66] | 3.43  [0.99, 5.26] | 0.75  [0.49, 1.47] | 0.85  [0.50, 2.91] | 0.08  [0.01, 0.28] | 0.54  [0.47, 0.76] | 0.08  [0.01, 0.27] | 0.16  [0.00, 0.33] |
| $D_{lungs}^{mean}$ | 30.34  [4.71, 51.73] | 21.03  [15.70, 27.30] | 68.30  [64.25, 77.93] | 4.75  [0.29, 22.62] | 27.01  [21.42, 33.04] | 19.48  [16.17, 26.76] | 10.98  [8.89, 18.39] | 4.73  [2.20, 15.56] |

median, [min, max]

Table S2 Absolute percent differences of $D^{mean}$ in VOIs using partition model and VSV methods compared with MC in patient data of Taipei Veterans General Hospital (n=14).

| Methods | Partition model | LED | LiK | LiLuK | LiKD | LiCK | LiLuKD | LiCKLuKD |
| --- | --- | --- | --- | --- | --- | --- | --- | --- |
| $D_{NL}^{mean}$ | 1.59  [0.10, 3.20] | 0.44  [0.07, 3.11] | 1.93  [0.61, 3.90] | 3.19  [1.70, 6.65] | 0.12  [0.05, 0.58] | 0.75  [0.47, 2.76] | 0.17  [0.01, 0.59] | 0.23  [0.10, 0.59] |
| $D_{TL}^{mean}$ | 1.97  [0.06, 4.24] | 2.21  [0.51, 6.34] | 1.78  [0.08, 3.06] | 2.44  [0.78, 3.37] | 0.35  [0.04, 5.56] | 0.86  [0.55, 5.64] | 0.33  [0.01, 5.56] | 0.46  [0.14, 5.10] |
| $D_{lungs}^{mean}$ | 9.29  [3.55, 51.02] | 17.22  [3.64, 35.72] | 73.94  [65.24, 79.02] | 26.65  [6.94, 35.12] | 22.21  [11.52, 41.68] | 15.81  [4.45, 30.79] | 7.38  [0.21, 17.17] | 5.09  [0.98, 7.79] |

median, [min, max]

Table S3 Absolute percent differences of DVH dosimetrics using VSV methods compared with MC in patient data of University of Michigan Deep Blue Data sharing repository (n=6).

| Methods | LED | LiK | LiLuK | LiKD | LiCK | LiLuKD | LiCKLuKD |
| --- | --- | --- | --- | --- | --- | --- | --- |
| $V_{NL, 70 Gy}$ | 1.19  [0.21, 4.18] | 0.78  [0.39, 1.12] | 1.83  [0.69, 4.50] | 0.23  [0.05, 0.64] | 0.24  [0.13, 0.40] | 0.25  [0.09, 0.64] | 0.10  [0.02, 0.42] |
| $V_{TL, 200 Gy}$ | 0.54  [0.00, 17.65] | 0.85  [0.00, 5.88] | 0.85  [0.00, 5.88] | 0.85  [0.00, 5.88] | 0.00  [0.00, 0.89] | 0.85  [0.00, 5.88] | 0.33  [0.00, 0.89] |
| $V_{lungs, 13 Gy}$ | 9.85  [0.15, 62.75] | 70.33  [65.84, 96.92] | 9.78  [0.91, 55.82] | 6.96  [3.42, 63.27] | 6.82  [2.58, 64.46] | 5.11  [4.61, 50.06] | 1.15  [0.71, 47.90] |
| $V_{lungs, 5 Gy}$ | 4.57  [0.55, 11.52] | 65.65  [60.69, 94.88] | 5.01  [0.78, 13.32] | 2.86  [0.43, 14.81] | 3.53  [2.79, 11.85] | 2.01  [0.04, 11.71] | 2.12  [1.06, 7.46] |

median, [min, max]

Table S4 Absolute percent differences of DVH dosimetrics using VSV methods compared with MC in patient data of Taipei Veterans General Hospital (n=14).

| Methods | LED | LiK | LiLuK | LiKD | LiCK | LiLuKD | LiCKLuKD |
| --- | --- | --- | --- | --- | --- | --- | --- |
| $V_{NL, 70 Gy}$ | 2.20  [0.06, 5.98] | 1.50  [0.46, 4.94] | 2.82  [0.45, 8.44] | 0.19  [0.07, 0.64] | 0.29  [0.00, 0.72] | 0.27  [0.01, 0.64] | 0.34  [0.06, 0.64] |
| $V_{TL, 200 Gy}$ | 1.04  [0.00, 39.73] | 2.31  [0.00, 26.53] | 2.64  [0.00, 26.53] | 0.36  [0.00, 4.08] | 0.39  [0.00, 3.20] | 0.37  [0.00, 4.08] | 0.40  [0.00, 2.33] |
| $V_{lungs, 13 Gy}$ | 15.06  [3.36, 55.10] | 76.23  [61.50, 87.08] | 25.34  [1.13, 59.16] | 14.39  [0.04, 48.73] | 14.72  [1.42, 44.35] | 6.99  [0.51, 33.49] | 7.19  [0.46, 30.42] |
| $V_{lungs, 5 Gy}$ | 19.20  [6.33, 35.67] | 69.29  [55.77, 85.53] | 16.03  [2.77, 36.84] | 13.48  [0.29, 28.11] | 12.94  [0.34, 27.73] | 4.89  [0.33, 15.87] | 5.73  [0.15, 16.76] |

median, [min, max]
